# Scientific contribution of the Brazilian CNPq Research Productivity fellows in dentistry

**DOI:** 10.1590/1807-3107bor-2024.vol38.0125

**Published:** 2024-12-09

**Authors:** Valéria Gonzaga Botelho de Oliveira, Árlen Almeida Duarte de Sousa, Fabrício Emanuel Soares de Oliveira, Daniella Reis Barbosa Martelli, Eduardo Araújo, Ricardo Della Coletta, Hercílio Martelli

**Affiliations:** (a) Universidade Estadual de Montes Claros – Unimontes, Postgraduate Program in Health Sciences, Montes Claros, MG, Brazil.; (b) Universidade Estadual de Montes Claros – Unimontes, Department of Educational Methods and Techniques, Montes Claros, MG, Brazil.; (c) Universidade Estadual de Montes Claros – Unimontes, Dental School, Oral Pathology and Oral Medicine, Montes Claros, MG, Brazil.; (d) Universidade Federal de Minas Gerais – UFMG, School of Medicine, Department of Pediatrics, Belo Horizonte, MG, Brazil.; (e) Universidade Estadual de Campinas – Unicamp, School of Dentistry, Department of Oral Diagnosis, Piracicaba, SP, Brazil.

**Keywords:** Research Personnel, Dentistry

## Abstract

This study evaluated the scientific contribution of Brazilian CNPq Research Productivity fellows (PQ) in Dentistry by areas of activity. This cross-sectional study included 217 active PQ who were grouped into six groups: (1) Biomaterials, Prosthodontics, and Restorative Dentistry; (2) Public Health and Epidemiology; (3) Oral Pathology, Stomatology, and Dental Radiology; (4) Pediatric Dentistry and Child Health; (5) Dental Clinic (Periodontics, Endodontics, Orthodontics, Oral Surgery, and Implantology), and (6) Basic Areas (Histology, Biochemistry, Molecular Biology, Physiology, Microbiology, Immunology, and Pharmacology). The PQ were predominantly male (n = 122; 56.2%), received level 2 scholarships (n = 121; 55.8%), and performed research in the Southeast region of Brazil (n = 160; 73.73%). Regarding supervision of undergraduate, master's, and PhD students, both during their entire careers and in the last 5 years, the highest average was observed for PQs in the field of Public Health and Epidemiology, the only area with higher average supervision of master's than that of undergraduate and PhD students. PQ in Public Health and Epidemiology had the highest average number of papers published over their career and in the last 5 years, followed by PQ in Pediatric Dentistry and Child Health and Dental Clinic. The high productivity of PQ is demonstrated by modern research performance indicators. Their scientific publications are indexed in bibliometric databases such as WoS, Scopus, and SciELO. Addtionally, highlighted among the PQ was the time since initiation of their scientific careers and master's and doctoral candidates trained.

## Introduction

Brazilian scientific production has experienced significant growth and increased international visibility over the past two decades. This has influenced the country's position in the world ranking in the number of publications in journals indexed in the Scopus database.^1^ However, such growth indicators require consistent financial investments to guarantee continuous quantitative and qualitative productivity.^2^

During the COVID-19 pandemic, the role of science for Brazilian citizens became even more evident, particularly in the country's response to the enormous pandemic challenges. Despite these challenges, scientific production in Brazil has been highlighted, together with that of China, India, South Korea, Turkey, and Iran.^3,4^

Brazil has two main national science funding agencies: the Coordination for the Improvement of Higher Education Personnel (Capes) (www.capes.gov.org) and the National Council for Scientific and Technological Development (CNPq) (www.cnpq.br). Both agencies were established in the 1950s. CAPES provides financial support and evaluates student performance in postgraduate courses (https://sucupira.capes.gov.br/sucupira/), whereas CNPq is devoted primarily to research funding. It also offers a research grant called the Research Productivity fellowship (termed PQ in Brazil). These researchers are currently classified into three categories (1, 2, and senior). Category 1 is further subdivided into four levels: 1A, 1B, 1C, and 1D. This scholarship implemented in the 1970s is for researchers with notable scientific contributions in their respective areas (https://www.gov.br/cnpq/pt-br/acesso-a-informacao/bolsas-e-auxilios/copy_of_modalidades).

The CNPq research productivity scholarship is aimed at Brazilian or foreign researchers residing in Brazil with recognized scientific and technological merits. The main objective of this scholarship is to encourage high-quality scientific and technological production, in addition to encouraging the training of qualified students. To be awarded a PQ scholarship, researchers must go through a rigorous evaluation of their scientific production, which includes an analysis of their curriculum vitae, publications, and mentorship activities, among other criteria. The CNPq generally holds an annual call for the research productivity scholarship via announcements posted on its official website and through other communication channels. During this period, researchers interested in competing for the scholarship must submit their applications, following the CNPq requirements and criteria.^5^ In 2023 alone, 3,935 of the 12,258 researchers applied for the CNPq productivity grant were awarded. In the last five years (2019-2023), 226 dentistry researchers were awarded a PQ grant.^6^ In 2023, the applicants and recipients were 189 and 42, respectively. Furthermore, Brazil has 166 *Stricto sensu* courses in Dentistry, including 100 programs (master's and PhD).^7^

Previous studies have highlighted the importance of PQ performance and contributions in areas such as Medicine,^8–10^ Dentistry,^11^ and Physical Therapy.^12^ Brazil stands out worldwide in key areas of research, such as Tropical Medicine, Dentistry, Parasitology, and more recently, Zika virus and microcephaly.^13^ Assessing PQ contribution in Dentistry is fundamental to recognize and value the impact of their research on the development of new knowledge, generation of technologies, training of students, and advances in the area, ensuring recognition and encouragement of excellence in research. This cross-sectional study aimed to evaluate the contribution of PQ in Dentistry by areas of activity.

## Methods

This cross-sectional study included 217 active PQ from different areas of Dentistry (http://memoria2.cnpq.br/bolsistas-vigentes). The curriculum vitae of each selected researcher was acquired through the CNPq Lattes platform (https://lattes.cnpq.br/) and used to extract the required information for the study, which was grouped into three sets of variables: researcher characteristics, scientific publications, and mentorship duties.

From the profile of each researcher, we collected information about their sex and geographic region in which they performed their professional activities, their scholarship level (1A, 1B, 1C, 1D, and 2), and the H-index. The duration of the PQ level 1A grant is 60 months; levels 1B, C, and Dare of 48 months; and level 2 is 36 months. The CNPq recently changed the identification of scholarship levels, removing number 1 from the initial categories (A-D) and replacing level 2 with E^14^. However, for the purpose of this study, the previous classification was adopted, as it was the method used during study time and data base construction.

The PQ were divided into six groups for comparison: (1) Biomaterials, Prosthesis, and Restorative Dentistry; (2) Public Health and Epidemiology; (3) Oral Pathology, Stomatology, and Dental Radiology; (4) Pediatric Dentistry and Child Health; (5) Dental Clinic (Periodontics, Endodontics, Orthodontics, Oral Surgery, and Implantology); and (6) Basic Areas (Histology, Biochemistry, Molecular Biology, Physiology, Microbiology, Immunology, and Pharmacology). Information on scientific publications included the number of scientific articles published throughout the PQ's career (period defined between the first scientific publication until December 2022) and in the last five years (2018-2022) and the total number of citations in the SciELO (https://scielo.org/), Scopus (https://www.scopus.com/), and Publons (https://www.webofscience.com) databases. In the dimension of mentorship, the information obtained was about scientific initiation of their career and number of master's and PhD students trained throughout their career and in the last five years (2018–2022).

The *Statistical Package for the Social Sciences* ^®^ 26.0 were used to analyze the data. Categorical variables (sex, fellowship level, and Brazilian region) were analyzed using relative and absolute frequencies. For numerical variables, means and 95% confidence intervals (CIs) were used (average of guidance, scientific articles, and citations), along with medians, percentiles, and interquartile range (IQR) minimum and maximum values, in addition to identifying outliers using a boxplot (H-index). This study used public and secondary data and did not require submission to a Research Ethics Committee for ethical approval.

## Results

**Figure 1:**
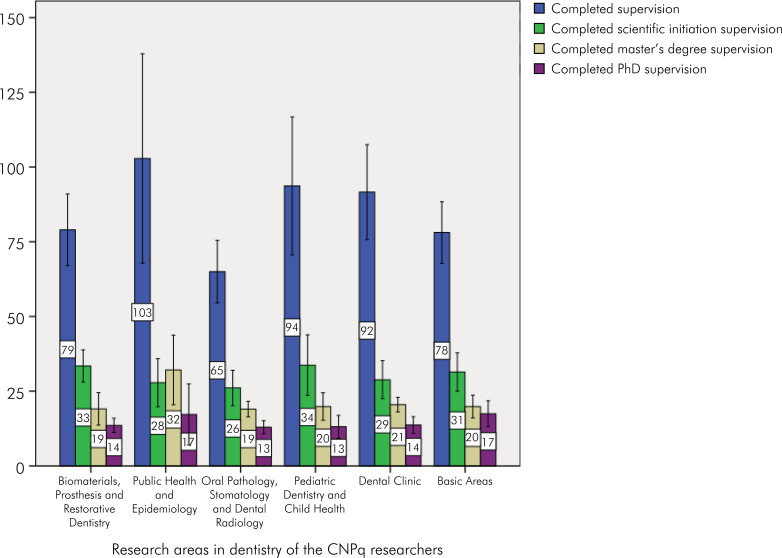
Average career supervision for CNPq researchers in the field of dentistry.

Among the 217 PQ, the majority were male (n = 122; 56.22%) and classified as level 2 researchers (n = 126; 58.06%). The Brazilian region with the highest prevalence of PQ was the Southeast (73.73%). The North region did not have any researchers (Table). Regarding the supervision of undergraduate, master's, and PhD students in the last five years (2018–2022), the highest average was observed among PQ in the field of Public Health and Epidemiology (Mean = 102.88; 95%CI: 67.83–137.92), which was also the only area with a higher average supervision of master's students than that of undergraduate and PhD students. In all other fields, the pattern showed a higher average for undergraduate supervision, followed by master's and PhDs (Figure 1).

**Table 1:** Characterization of CNPq researchers in dentistry areas (n = 217).

Research areas		Biomaterials, prosthesis, and restorative dentistry	Public health and epidemiology	Oral pathology, stomatology, and dental radiology	Pediatric dentistry and child health	Dental clinic	Basic areas	Total
		n (%)	n (%)	n (%)	n (%)	n (%)	n (%)	
Sex								
	Female	30 (49.2)	3 (37.5)	14 (41.2)	11 (42.3)	26 (43.3)	11 (39.3)	95 (43.8)
	Male	31 (50.8)	5 (62.5)	20 (58.8)	15 (57.7)	34 (56.7)	17 (60.7)	122 (56.2)
Level of scholarship								
	1A	7 (11.5)	0 ()	2 (5.9)	2 (7.7)	9 (15)	1 (3.6)	21 (9.7)
	1B	3 (4.9)	2 (25)	6 (17.6)	2 (7.7)	6 (10)	5 (17.9)	24 (11.1)
	1C	7 (11.5)	0 ()	3 (8.8)	3 (11.5)	6 (10)	5 (17.9)	24 (11.1)
	1D	5 (8.2)	2 (25)	7 (20.6)	5 (19.2)	4 (6.7)	3 (10.7)	26 (12)
	2	39 (63.9)	4 (50)	15 (44.1)	14 (53.8)	35 (58.3)	14 (50)	121 (55.8)
	SR	0 (0)	0 (0)	1 (2.9)	0 (0)	0 (0)	0 (0)	1 (0.5)
Postdoctoral								
	Yes	37 (60.7)	7 (87.5)	22 (64.7)	19 (73.1)	33 (55)	24 (85.7)	142 (65.4)
	No	24 (39.3)	1 (12.5)	12 (7)	7 (26.9)	27 (45)	4 (14.3)	75 (34.6)
Brazilian regions								
	Midwest	1 (1.6)	0 (0)	2 (5.9)	2 (7.7)	1 (1.7)	1 (3.6)	7 (3.2)
	Northeast	6 (9.8)	2 (25)	9 (26.5)	1 (3.8)	4 (6.7)	2 (7.1)	24 (11.1)
	South	11 (18)	0 (0)	1 (2.9)	3 (11.5)	8 (13.3)	3 (10.7)	26 (12)
	Southeast	43 (70.5)	6 (75)	22 (64.7)	20 (76.9)	47 (78.3)	22 (78.6)	160 (73.7)

**Figure 2:**
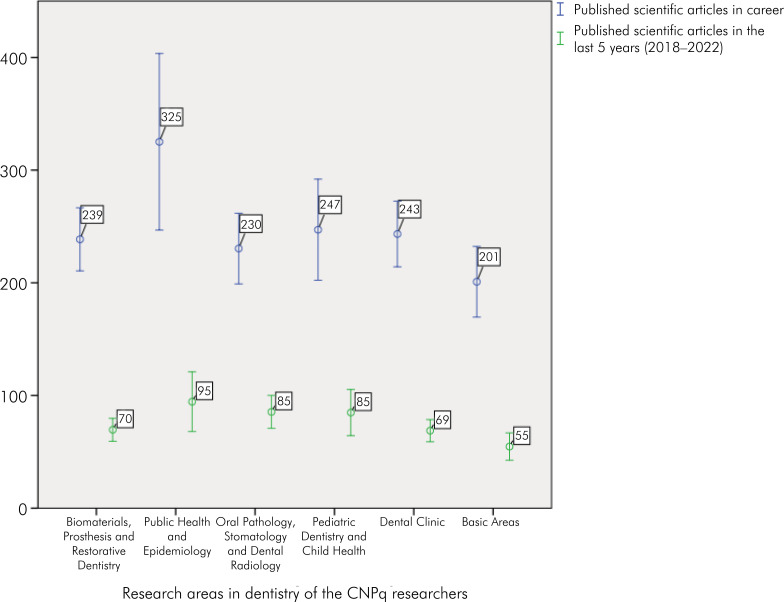
Average number of published scientific articles throughout the career and in the last 5 years of CNPq researchers in the field of dentistry.

The highest average of career-published papers was found for PQ in the field of Public Health and Epidemiology (Mean = 325.13; 95%CI: 246.79–403.46). Public Health and Epidemiology was also the area with the highest average number of publications in the last 5 years (Mean = 95; 95%CI: 68.04–120.96), followed by Pediatric Dentistry and Child Health and Dental Clinic (Figure 2).

**Figure 3:**
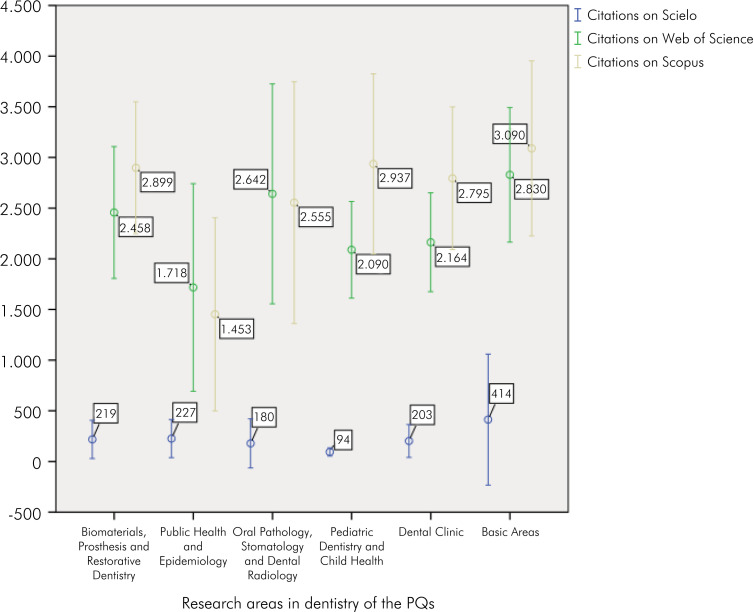
Average citations in databases (Scielo, Web of Science, and Scopus) for CNPq researchers in the field of dentistry.

Figure 3 depicts the average number of citations (with 95%CIs) in databases (SciELO, WoS, and Scopus). A higher average of citations was observed in the Web of Science and Scopus, with SciELO having a lower number. The Basic Areas obtained the highest average citations in all databases. The field of Pediatric Dentistry and Child Health had the second-highest average number of citations in Scopus, and in the Web of Science, the area that obtained the second-highest citation average was Oral Pathology, Stomatology, and Dental Radiology. In SciELO, the Basic Areas had the highest average, Pediatric Dentistry and Child Health had the lowest, and all other areas had citation averages close to 200 (Figure 3).

**Figure 4:**
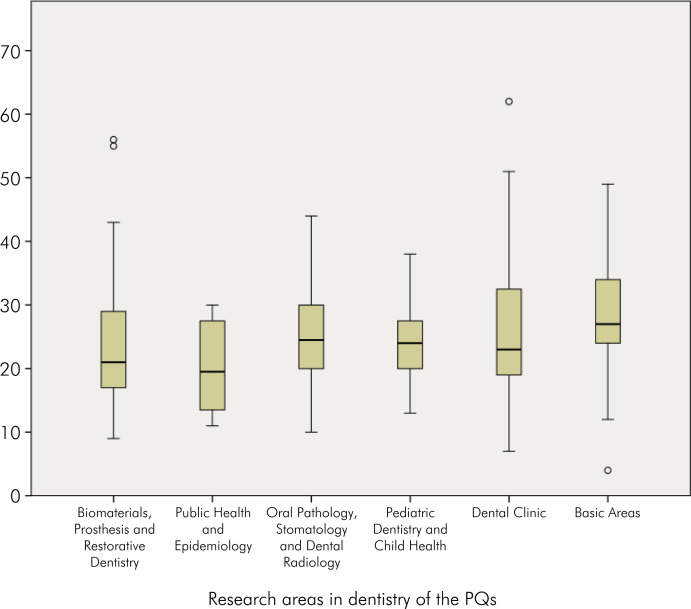
Boxplot of the H-index for CNPq researchers in the field of dentistry.

The Basic Areas were also notable concerning the H-index in Publons (WoS) compared to other areas, with the highest median of 27 (IQR, 17–29; range 4–49), followed by the areas of Oral Pathology, Stomatology, and Dental Radiology, and Pediatric Dentistry and Child Health, which had medians of 24.5 (IQR, 20–30; range 10–44) and 24 (IQR, 13–38; range 4–49), respectively (Figure 4).

## Discussion

The present study focused on evaluating the contribution of Brazilian CNPq researchers in Dentistry by area of expertise with regard to several scientific indicators. As expected, our analysis showed that overall, this group had high scientific productivity in several areas of academic activity in Dentistry. From a quality perspective, it is also noteworthy that most papers by these researchers published most papers in journals indexed in prominent databases, such as SciELO, WoS, and Scopus.

Our analysis showed a predominance of male researchers (1.28:1), although there are currently more female than male undergraduate and postgraduate students in Brazil.^15^ The predominance of male researchers in various areas of research is a complex and multifaceted phenomenon influenced by a series of historical, social, cultural, and structural factors.^16^ In this sense, Lariviere et al.^17^ have presented a bibliometric analysis confirming that gender inequalities persist in research output worldwide. In Brazil, fewer women have academic positions associated with higher income and higher academic prestige.^18^ In a recent study, it was observed that in the area of Pediatric Dentistry, there was a predominance of female researchers (66.7%), similar to the areas of education, physical therapy, and occupational therapy.^5,19^ Studies addressing funding for projects by the US National Institute of Dental and Craniofacial Research did not find significant differences between sexes,^20^ reporting parity between the sexes of the first and last authors.^21^

The geographical distribution of the analyzed CNPq researchers analyzed is not homogenous. One hundred and sixty (73.73%) resided in the Southeast of Brazil. No researchers were located in the North region. This grouping is also observed in other studies that describe the predominance of scholarship holders, mainly in the states of São Paulo and Minas Gerais,^22,23^ and the same pattern occurs in other research areas.^8,9,24^ Some indicators may help explain this predominance in the Southeast region. Of the 70 higher educational institutions offering postgraduate programs in Dentistry in Brazil, 31 (44.28%) are in the Southeast. Of the 167 master's and PhD programs, most are concentrated in the Southeast, and all postgraduate programs with excellent grades (6 and 7) are in this region (https://sucupira.capes.gov.br/sucupira/public/consultas/coleta/programa/quantitativos/quantitativoIes.jsf?areaAvaliacao=18&areaConhecimento=40200000).

Another important indicator in our analysis was the scientific production of this group of CNPq researchers, both throughout their careers and over the last five years. The publications of papers throughout their careers ranged from 201 (Basic Areas) to 325 (Public Health and Epidemiology), with a significant increase in publications in the last five years in all six areas. However, in the last five years, the area with the highest average number of publications was Public Health and Epidemiology. This increase in scientific production has also been observed in other areas such as Public Health and Chemistry.^25,26^ We believe that this quantitative increase in the productivity of researchers in dentistry is also correlated with the general increase in Brazilian scientific production and reflects the various incentive mechanisms that have been implemented by Brazilian research agencies.^27^ Among them is the improvement of the system for the evaluation of postgraduate programs. The Brazilian institution responsible for this evaluation is the CAPES, which prioritizes the number and quality of published articles when ranking Brazilian postgraduate programs. Another mechanism is the diverse modality of scholarships, including productivity in research scholarships, which promotes competition among peers, encouraging both the training of new researchers and the publication of scientific articles in renowned journals.

One dimension of CNPq researchers is participation in mentorship. In the present study, an important average of undergraduate, master's degree, and PhD student training was observed throughout the researchers’ careers and in the assessed five-year period. These results are similar to those of other studies from different areas involving scientific production and student training.^28–30^ The PQ also advised 6,641 undergraduate students conducting research (median of 28; range: 0–122), 4,373 master's students (median of 18; range: 4–171), and 3,061 PhD students (median of 11; range: 1–55). Regarding the quantity of undergraduate, master's, and PhD students supervised by the PQ in the last five years, the highest average was observed in the field of Public Health and Epidemiology, being the only area with a higher average of PQ supervising master's than undergraduate and PhD students.

Collectively, our analyses reveal that this group exhibits high scientific productivity. Notably, from a quality standpoint, a significant portion of the papers authored by these researchers were published in journals indexed in reputable databases, such as Web of Science and Scopus, with SciELO having the lowest number of citations. The Basic Areas had the highest average number of citations in all databases. The field of Pediatric Dentistry and Child Health had the second-highest average number of citations in Scopus, and in the Web of Science, the area that had the second-highest citation average was Oral Pathology, Stomatology, and Dental Radiology.

Among several indicators for the assessment of the research performance of scientists, the H-index, proposed by Hirsch,^31^ is intended to simultaneously measure the quality and sustainability of scientific output. The H-index is a single number that summarizes an author's research output and its impact. The index is based on the scientists’ most-cited papers and the number of citations that they have received in publications by other scientists.^32,33^ The H-index brings together the two dimensions of academic performance in one measure. These dimensions are productivity (number of publications) and visibility (number of citations). The H-index is insensitive to excessive productivity of publications receiving few or no citations and to authors having published comparatively few articles but receiving good visibility through numerous citations.^34^ In the present study, in general, the six groups of researchers analyzed showed a significant H-index, reflecting the quality of the scientific publications and their visibility. The three areas with the highest H-indices were Basic Areas (27; IQR, 17–29; range 4–49), Oral Pathology, Stomatology, and Dental Radiology (24.5; IQR, 20–30; range 10–44) and Pediatric Dentistry and Child Health (24; IQR, 13–38; range 4–49).

Several methodological considerations should be considered when evaluating the findings of this study. We believe that future studies can be improved by including the impact factor of journals as a covariate in the analysis. Another possible limitation of this study was the inclusion of several subfields of Dentistry in our analysis. Usually, research performance indicators, such as impact factors, exhibit significant variation according to the subject field.^35^ In general, fundamental and basic subject areas have higher average impact factors than specialized or applied ones. Perhaps a correction of the H-index for the duration of researcher's scientific activity needs to be applied.

Although the present study was conducted on the 217 PQ of the CNPq, it is important to highlight that this number is well below the density and impact of Brazilian dental research in the international scenario.^11,29,36,37^ The country has 166 *Stricto sensu* courses in various areas of Dentistry,^7^ in addition to a significant representation of dental research in events such as the Brazilian Society of Dental Research (https://www.sbpqo.org.br/). Although we were unable to specify the total number of Brazilian researchers in Dentistry, the advances and highlights of the PQ scholars are noticeable and influential.^36,38^

In light of our findings, we believe that the CNPq ranking system based mainly on journal impact factors is insufficient for distinguishing researchers with outstanding scientific production. Further studies addressing this issue may contribute to a more judicious distribution of resources among competing researchers.

## Conclusion

Our findings show that Research Productivity fellows in Dentistry who are recipients of CNPq productivity scholarships are a group with high scientific productivity, as ascertained by modern indicators of research performance. The data produced in this study contribute substantially to the dental field, boosting the advancement of scientific knowledge and highlighting the importance of the Brazilian dentistry area in the research scenario. However, a comparison with similar groups in other countries is difficult owing to the scarcity of studies in this field.

## References

[1] Science & Engineering Indicators Publications output: U.S. trends and international comparisons.

[2] Martelli H, Martelli DR, Silva ACS, Oliveira MC, Oliveira EA (2019). Brazil's endangered postgraduate system. Science.

[3] Silva ACS, Oliveira EA, Martelli H (2020). Coronavirus disease pandemic is a real challenge for Brazil. Front Public Health.

[4] Oliveira EA, Oliveira MC, Colosimo EA, Martelli DR, Silva AC, Martelli H (2022). Global scientific production in the pre-Covid-19 era: an analysis of 53 countries for 22 years. An Acad Bras Cienc.

[5] Conselho Nacional de Desenvolvimento Científico e Tecnológico (2023). Chamada CNPq/MCT/Mulheres n° 31/2023. Meninas nas ciências exatas, engenharias e computação.

[6] Conselho Nacional de Desenvolvimento Científico e Tecnológico (2024). Mapa de fomento em ciência, tecnologia e inovação. Bolsas e projetos vigentes.

[7] Ministério da Educação (BR) (2024). Plataforma Sucupira. Cursos avaliados e reconhecidos.

[8] Dias GP, Martelli DR, Costa SM, Andrade RS, Oliveira EA, Martelli H (2020). Scientific production of researchers from the Brazilian Council for Scientific and Technological Development (CNPq) in the neuroscience area. Rev Bras Educ Med.

[9] Oliveira EA, Ribeiro AL, Quirino IG, Oliveira MC, Martelli DR, Lima LS (2011). Profile and scientific production of CNPq researchers in cardiology. Arq Bras Cardiol.

[10] Sales GH, Martelli DR, Oliveira EA, Dias VO, Oliveira MC, Martelli H (2017). Evaluation on the scientific production in fields of medicine: a comparative study. Rev Bras Educ Med.

[11] Andrade RS, Martelli DR, Swerts MS, Oliveira EA, Martelli H (2018). Scientific production of the Brazilian Council for Scientific and Technological Development (CNPq) researchers in the field of Oral Medicine and Oral Pathology granted with a scientific productivity fellowship. Oral Surg Oral Med Oral Pathol Oral Radiol.

[12] Coury HJ, Mancini MC (2008). Representation of physical therapy and occupational therapy in CNPq. Braz J Phys Ther.

[13] Coordenação de Aperfeiçoamento de Pessoal de Nível Superior (2018). Proposta de aprimoramento do modelo de avaliação da PG. Documento Final da Comissão Nacional de Acompanhamento do PNPG 2011-2020.

[14] Conselho Nacional de Desenvolvimento Científico e Tecnológico (2023). Resolução-3/2023, de 17 de outubro de 2023. Altera a identificação dos níveis das bolsas das modalidades de produtividade do CNPq.

[15] Conselho Nacional de Desenvolvimento Científico e Tecnológico (2023). Mulheres na ciência: para cientistas, ascender na carreira ainda é um desafio.

[16] Keffer GM, Sousa AAD, Oliveira FE, Magalhães MJS, Oliveira EA, Martelli H (2024). Evaluation of Brazilian women's participation in the CNPQ in the field of medical research. Rev Bras Educ Med.

[17] Larivière V, Ni C, Gingras Y, Cronin B, Sugimoto CR (2013). Bibliometrics: global gender disparities in science. Nature.

[18] Moschkovich M, Almeida AM (2015). Gender inequalities in academic careers in Brazil. Dados.

[19] Souza AA, Brito AM, Alves SA, Vicente JVJ, Oliveira VGB, Martelli DR (2023). Scientific production of CNPq researchers in the areas of physical therapy and occupational therapy, Brazil. Fisioter Mov.

[20] Garcia MN, Tiano JP, Contreras O, Hildebolt CF, Horsford J, Stewart D (2020). Trends in academic dentistry and oral health research funding by gender. JDR Clin Trans Res.

[21] Shah SG, Dam R, Milano MJ, Edmunds LD, Henderson LR, Hartley CR (2021). Gender parity in scientific authorship in a National Institute for Health Research Biomedical Research Centre: a bibliometric analysis. BMJ Open.

[22] Freire RS, Oliveira EA, Silveira MF, Martelli DR, Oliveira MC, Martelli H (2013). Profile of researchers of the National Council for Scientific and Technological Development in the fields of Physiotherapy and Occupational Therapy. RBPG.

[23] Melo NG, Cunha IA, Alves JF, Santos AL, Nogueira AP, Lima BC (2021). Perfil de formação e produção científica do fisioterapeuta pesquisador no Brasil. Fisioter Pesqui.

[24] Oliveira MC, Martelli DR, Quirino IG, Colosimo EA, Silva AC, Martelli H (2014). Profile and scientific production of the Brazilian Council for Scientific and Technological Development (CNPq) researchers in the field of hematology/oncology. Rev Assoc Med Bras.

[25] Barata RB, Goldbaum M (2003). [A profile of researchers in public health with productivity grants from the Brazilian National Research Council (CNPq)]. Cad Saude Publica.

[26] Santos NC, Candido LF, Kuppens CL (2010). Produtividade em pesquisa do CNPq: analise do perfil dos pesquisadores da química. Quim Nova.

[27] Leite P, Mugnaini R, Leta J (2011). A new indicator for international visibility: exploring Brazilian scientific community. Scientometrics.

[28] Oliveira EA, Colosimo EA, Martelli DR, Quirino IG, Oliveira MC, Silva LS (2012). Comparison of Brazilian researchers in clinical medicine: are criteria for ranking well-adjusted?. Scientometrics.

[29] Andrade RS, Martelli DR, Almeida OP, Lopes MA, Swerts MS, Pires FR (2018). Brazilian scientific production in oral medicine and oral pathology. Oral Surg Oral Med Oral Pathol Oral Radiol.

[30] Rodrigues LO, Gouvea MM, Marques FC, Mourao SC (2017). Overview of the scientific production in the Pharmacy area in Brazil: profile and productivity of researchers granted with fellowships by the National Council for Scientific and Technological Development. Scientometrics.

[31] Hirsch JE (2005). An index to quantify an individual's scientific research output. Proc Natl Acad Sci USA.

[32] Burrell QL (2007). Hirsch's h-index: a stochastic model. J Informetrics.

[33] Panaretos J, Malesios C (2009). Assessing scientific research performance and impact with single indices. Scientometrics.

[34] Boell SK, Wilson CS (2010). Journal impact factors for evaluating scientific performance: use of h-like indicators. Scientometrics.

[35] Glanzel W, Schubert A (2003). A new classification scheme of science fields and subfields designed for scientometric evaluation purposes. Scientometrics.

[36] Lima LC, Bernardino VM, Prata IM, Lopes RT, Silva SE, Sousa ML (2022). Profile of brazilian research productivity grant holders with a background in pediatric dentistry. Braz Dent J.

[37] Farias LC, Trezena S, de Souza HC, Coletta RD, Martelli H (2024). Internationalization of the Brazilian groups dedicated to oral pathology and oral medicine. Oral Dis.

[38] Gomes D, Agnoletto IG, Souza ML, Spiger V, Jakymiu JR, Fujii EC (2017). A produção científica da Odontologia e a Agenda Nacional de Prioridades de Pesquisa em Saúde. Rev ABENO.

